# Developing Pulmonary Vasculopathy in Systemic Sclerosis, Detected with Non-Invasive Cardiopulmonary Exercise Testing

**DOI:** 10.1371/journal.pone.0014293

**Published:** 2010-12-13

**Authors:** Daniel Dumitrescu, Ronald J. Oudiz, George Karpouzas, Arsen Hovanesyan, Amali Jayasinghe, James E. Hansen, Stephan Rosenkranz, Karlman Wasserman

**Affiliations:** 1 Los Angeles Biomedical Research Institute, Harbor-UCLA Medical Center, Torrance, California, United States of America; 2 Division of Respiratory and Critical Care Medicine and Physiology, Klinik III fuer Innere Medizin, Herzzentrum der Universitaet zu Koeln, Cologne, Germany; 3 Division of Cardiology, Klinik III fuer Innere Medizin, Herzzentrum der Universitaet zu Koeln, Cologne, Germany; 4 Division of Rheumatology, Klinik III fuer Innere Medizin, Herzzentrum der Universitaet zu Koeln, Cologne, Germany; 5 Klinik III fuer Innere Medizin, Herzzentrum der Universitaet zu Koeln, Cologne, Germany; University of Giessen Lung Center, Germany

## Abstract

**Background:**

Patients with systemic sclerosis (SSc) may develop exercise intolerance due to musculoskeletal involvement, restrictive lung disease, left ventricular dysfunction, or pulmonary vasculopathy (PV). The latter is particularly important since it may lead to lethal pulmonary arterial hypertension (PAH). We hypothesized that abnormalities during cardiopulmonary exercise testing (CPET) in patients with SSc can identify PV leading to overt PAH.

**Methods:**

Thirty SSc patients from the Harbor-UCLA Rheumatology clinic, not clinically suspected of having significant pulmonary vascular disease, were referred for this prospective study. Resting pulmonary function and exercise gas exchange were assessed, including peakVO_2_, anaerobic threshold (*AT*), heart rate- VO_2_ relationship (O_2_-pulse), exercise breathing reserve and parameters of ventilation-perfusion mismatching, as evidenced by elevated ventilatory equivalent for CO_2_ (VE/VCO_2_) and reduced end-tidal pCO_2_ (P_ET_CO_2_) at the *AT*.

**Results:**

Gas exchange patterns were abnormal in 16 pts with specific cardiopulmonary disease physiology: Eleven patients had findings consistent with PV, while five had findings consistent with left-ventricular dysfunction (LVD). Although both groups had low peak VO_2_ and *AT*, a higher VE/VCO_2_ at *AT* and decreasing P_ET_CO_2_ during early exercise distinguished PV from LVD.

**Conclusions:**

Previously undiagnosed exercise impairments due to LVD or PV were common in our SSc patients. Cardiopulmonary exercise testing may help to differentiate and detect these disorders early in patients with SSc.

## Introduction

Dyspnea on exertion, fatigue, and reduced exercise tolerance are common symptoms in patients with systemic sclerosis (SSc). These symptoms can often be explained by involvement of the musculoskeletal system, lungs, heart, chest wall, and/or pulmonary vasculature, in isolation or combination. Patients with SSc are at particular risk for developing pulmonary vasculopathy (PV) leading to pulmonary arterial hypertension (PAH). Untreated, PAH results in right ventricular failure, and early death [1].

PV impairs dilatation of affected pulmonary blood vessels, impeding pulmonary blood flow during exercise. This eventually leads to pulmonary hypertension and exercise intolerance. Initially, the degree of exercise limitation is determined by the ability of the right ventricle to hypertrophy and maintain adequate blood flow through the lungs. At this stage, pulmonary hypertension might only be visible during exercise [2], [3]. Over time, vasculopathy progresses and the right ventricular reserve fails to meet the pulmonary blood flow required for the increased O_2_ demand of exercise, leading to exertional dyspnea and fatigue and physical signs of pulmonary hypertension.

Early detection of PV may be desirable since timely therapeutic intervention improves outcomes in experimental models [4], [5]. Additionally, treatment of patients with early PAH can delay clinical worsening [6]. Pulmonary vasculopathy develops unevenly in the lungs. Thus, abnormal gas exchange findings characteristic of ventilation-perfusion mismatching, is an early abnormality during cardiopulmonary exercise testing (CPET) [7].

The gas exchange abnormalities during CPET in patients with PV reflect hypoperfusion of well-ventilated acini. Thus, ventilation (VE) is high compared to relatively low CO_2_ output (VCO_2_) and reduced end-tidal PCO_2_ (P_ET_CO_2_), manifesting hypoperfusion of well-ventilated lung. In this study, we performed CPET in a group of referred SSc patients, without previously known or suspected PV. We expected that these patients would display heterogeneous gas exchange patterns during exercise, which cannot be explained by resting measurements alone. We hypothesized that we would find characteristic gas exchange patterns that would enable us to discriminate between the different causes of exercise intolerance, based on the exercise pathophysiology. We hypothesized that some of the patients would show gas exchange patterns during exercise that are characteristically found in patients with overt pulmonary vascular pathophysiology.

## Methods

### Ethics statement

This study was conducted in accordance with Good Clinical Practices and the current version of the revised Declaration of Helsinki [8]. The local Los Angeles Biomedical Research Institutional Review Board approved the protocol. A written informed consent was obtained from each patient prior to enrollment.

### Study population

We prospectively screened 32 SSc patients referred from the Rheumatology Clinic at Harbor-UCLA Medical Center for CPET in order to determine if they had evidence of PV. Prior to referral, all patients had chest X-rays and/or high-resolution chest CT-scans. All patients had echocardiography with estimation of pulmonary artery pressure (PAP) prior to referral. Patients with estimated systolic PAP >35 mmHg, were excluded.

All patients had been diagnosed with SSc according to the criteria of the American College of Rheumatology (ACR) [9]. One patient refused to perform CPET and another could not perform CPET because of joint stiffness. Thus, thirty patients performed CPET.

### Evaluations

#### 6-minute walk test

All patients performed an unencouraged, standardized 6-minute walk test (6MWD), at least one hour before or after CPET [10].

#### Pulmonary function testing

Total lung capacity (TLC), forced vital capacity (FVC), forced expired volume in one second (FEV_1_), diffusing capacity for carbon monoxide (DL_CO_) and alveolar volume (VA) were all measured as part of CPET and are expressed as percent predicted.

#### Assessment of restrictive lung disease

Restrictive lung disease was assessed by a combination of resting pulmonary function tests (PFTs), including diffusion capacity for carbon monoxide (DL_CO_), by Chest X-ray (CXR) and by high-resolution computed tomography (HRCT). An HRCT was performed if there were abnormalities in PFTs or CXR. An HRCT was not performed in patients with normal PFTs and a normal CXR, or a definite diagnosis of ILD based on these two measurements.

The available HRCT-scans in patients with suspected ILD (20 out of 30) were analyzed for signs of pulmonary venous occlusive disease (PVOD). Main characteristics were enlarged mediastinal lymph nodes, alveolar hemorrhage, centrilobular ground glass opacities and septal lines on HRCT.

#### Cardiopulmonary exercise testing

CPET was performed with upright cycling on a stationary cycle ergometer. The exercise protocol consisted of 3 minutes of rest and 3 minutes of unloaded cycling, followed by an incremental work rate between 5 and 15 watts per minute up to the patients' maximum tolerance, then 3 minutes of recovery. Gas exchange was measured breath-by-breath during the test, using a MedGraphics CPX-Ultima gas exchange system (Medical Graphics Corporation, St. Paul, Minnesota). Equipment was calibrated as previously described [11]. ECG and pulse oximetry were continuously monitored and blood pressure was measured every two minutes. Minute ventilation (VE), heart rate (HR), VO_2_/HR, VO_2_, VCO_2_, VCO_2_ vs VO_2_, VE/VO_2_, VE/VCO_2_, tidal volume (VT) vs VE, end-tidal PO_2_ (P_ET_O_2_) and PCO_2_ (P_ET_CO_2_) and the respiratory exchange ratio (RER) were averaged every 10 seconds. The anaerobic threshold (*AT*) was determined from gas exchange, by the V-slope method as previously described [12], in all patients. The *AT* was derived from a plot with VO_2_ (x-axis) and VCO_2_ (y-axis) on equal axis scaling, and was recognized as the point where VCO_2_ started to increase faster than VO_2_. *AT* prediction was performed as previously described [13], [14]. The other key variables were calculated and plotted as previously described [15], [16]. All studies were independently reviewed by two authors (DD and KW). Disagreements were adjudicated after review by a third author (JH), and consensus agreement among all three.

### Categorizing Exercise Impairment

**Figure 1:**
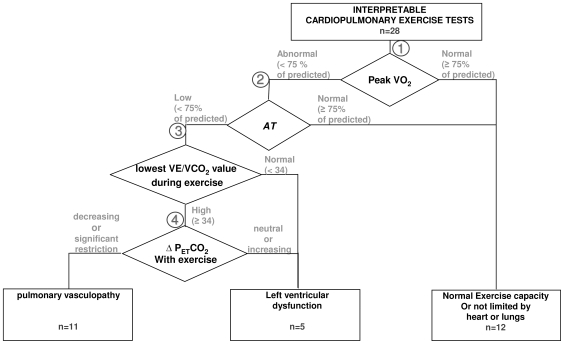
Categorizing referred SSc patients with normal and reduced exercise capacity, using cardiopulmonary exercise testing. Exercise intolerance was attributed to left ventricular dysfunction or pulmonary vascular disease. Normal is defined as either: a) normal in all cardiovascular and ventilatory aspects of exercise gas exchange, including normal ventilation-perfusion matching and normal peak VO_2_, or b) reduced peak VO_2_ with normal *AT* and no gas exchange abnormalities suggestive of heart, lung or pulmonary vascular disease. Diamonds (branch-points) address specific data: *Branch-point 1:* Right branch: If the peak VO_2_ is ≥75% of predicted with normal VE/VCO_2_ and P_ET_CO_2_ @ *AT* and non-ventilatory limitation, the patient is considered to have normal heart and lung function. Left branch includes all with peak VO_2_ <75%. *Branch-point 2:* If the *AT* is normal and ventilation-perfusion matching and lung mechanics are normal (right branch), the patient is considered to be limited by poor effort and not limited by heart or lung disease. If the *AT* is reduced (left branch), the patient is likely to have left ventricular dysfunction or pulmonary vasculopathy. *Branch-point 3:* The VE/VCO_2_ @*AT* was used to assess matching of ventilation to perfusion. All patients with pulmonary vasculopathy would have ventilation/perfusion mismatching and an elevated VE/VCO_2_. A cut-off value of ≥34 was selected. If not elevated, they were considered to have left ventricular dysfunction. *Branch point 4:* P_ET_CO_2_ usually increases from the beginning of exercise to the *AT* in patients with normal cardiopulmonary function and patients with left ventricular dysfunction (right branch). However, it usually decreases in patients with pulmonary arterial hypertension (left branch). Nine of the 11 patients classified as pulmonary vasculopathy had a decreasing P_ET_CO_2_. Two had either no change or increasing P_ET_CO_2_ from the start of exercise to the *AT*, possibly due to lung restriction. However, they hyperventilated above their *AT*. If the patient had moderate to severe restriction and marked decrease in DL_CO_, this signified interstitial lung disease with pulmonary vasculopathy.

Patients with known severe heart or lung disease limiting exercise, or individuals with known PAH, were not referred by the Rheumatologists. An additional two patients with uninterpretable cardiopulmonary exercise test results were not included in the analysis (2 of 30 patients). The first patient stopped during the unloaded cycling phase due to joint pain. The second patient had a very noisy and chaotic breathing pattern. For both of these patients, peak VO_2_, the *AT* and VE/VCO_2_ at the *AT* could not be accurately determined. Thus, 28 patients were available for analysis. Figure 1 presents the algorithm utilized. Disagreement in the blinded interpretation of the CPET studies occurred in 2 of 28 interpretable cases. Agreement was reached in these two cases by review of a third author. The normal category included those with a normal peak VO_2_, normal anaerobic threshold (*AT*) normal ventilation-perfusion matching, and no exercise-induced hypoxemia (6 of 28 patients). The normal category also included six patients with a reduced peak VO_2_, but with normal *AT*, no abnormality in ventilation-perfusion matching or exercise-induced hypoxemia and an RER at peak exercise below 1.0, indicating submaximal effort. These patients were categorized as not being limited by heart or lung disease. Thus, 12 of the 28 patients were categorized as normal.

Patients were categorized in the left ventricular dysfunction (LVD) group if they had a reduced peak VO_2_, *AT*, peak O_2_-pulse and Δ VO_2_/ΔWR - but without ventilation-perfusion mismatch or exercise-induced hypoxemia or RER at peak exercise <1.0. (5 of 28 patients were in this category).

Patients were categorized in the PV group if they had a reduced peak VO_2_ and *AT*, reduced peak O_2_-pulse and ΔVO_2_/ΔWR, and ventilation-perfusion mismatch (elevated VE/VCO_2_ at the *AT* or at the ventilatory compensation point (VCP) following AT). In addition, based on prior research [17], [18], suspected PV was separated from LVD by a decreasing P_ET_CO_2_ from the start of exercise to *AT* (9 of 28 patients were in this category), in contrast to an increasing P_ET_CO_2_ in LVD and normal subjects. Two other patients showed rising P_ET_CO_2_ during exercise but were classified as suspected PV secondary to their restrictive lung disease with parallel loss of pulmonary capillary volume (low TLC and DLCO with normal FEV_1_/FVC), however breathing reserve was thought to be adequate without mechanical ventilatory limitation at peak exercise. This is based on a prior study [19] showing that lung restriction from pulmonary fibrosis, before functional lung restriction, is accompanied by exercise limiting PV.

### Statistical analysis

A total of 28 of the SSc patients referred, with interpretable CPET studies, were analyzed; they were divided into 3 major categories: normal, LVD and PV, as described above. Continuous variables are expressed as mean ± SD. The three groups were individually compared to each other. Differences were analyzed using one way ANOVA, followed by Holm-Sidak testing for multiple comparisons. Nominal data were analyzed by Chi-square test for multiple groups. In all cases, a p value <0.05 was considered statistically significant.

## Results

Table 1 shows the demographics according to diagnostic category. All patients tolerated CPET well, and there were no adverse events.

**Table 1:** Demographics for each exercise diagnosis in 30 scleroderma patients.

	Not interpretable exercise test results(n = 2)	Normal exercise capacity (NL)(n = 12)	Left Ventricular Dysfunction(LVD)(n = 5)	Pulmonary vasculopathy(PV)(n = 11)	NL vs. LVD		p-valueNL vs. PV		LVD vs. PV
**M/F**	0/2	2/10	2/3	1/10					
**Limited/diffuse SSc**	2/0	9/3	3/2	9/2					
**NYHA Class I**	1/2	6/12	4/5	2/11					
**NYHA Class II**	1/2	6/12	1/5	8/11					
**NYHA Class III**	0/2	0/12	0/5	1/11					
**Age (years)**	51±1	52±7	41±11	49±14	n/s (p = 0.31)				
**BMI (kg/m^2^)**	28.2±7.2	28.5±7.7	27.0±3.9	26.4±5.7	n/s (p = 0.73)				
**ACA positive**	1/2	5/12	2/5	2/11	n/s (p = 0.44)				
**Scl-70 positive**	1/2	3/12	0/5	3/11	n/s (p = 0.30)				

ACA  =  anti-centromer antibodies.

Scl-70  =  DNA-topoisomerase I antibodies.

### Gas Exchange Patterns

**Figure 2:**
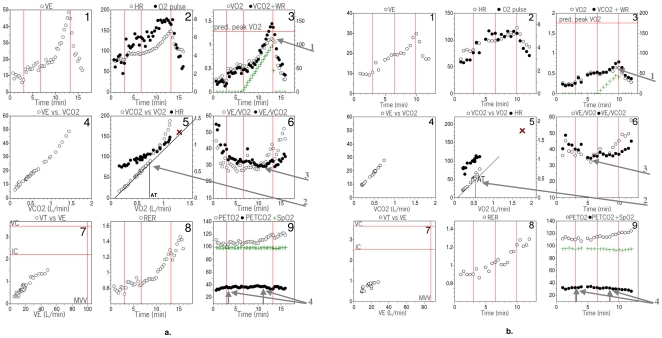
Gas exchange response to exercise in two SSc patients. Nine panel plots of a patient with normal exercise performance (Fig. 2a) and one with pulmonary vasculopathy (Fig. 2b). The protocol consisted of a 3-minute resting period, followed by 3 minutes of very-low-level cycle exercise, and then increasing cycle workload to the patient's maximum tolerance. Points are 20-second averages. Panel 1 is plot of ventilation against time. Panel 2 is plot of heart rate and O_2_-pulse against time. Panel 3 is plot of O_2_ uptake (VO_2_), CO_2_ output (VCO_2_) and work rate against time. Panel 4 is plot of minute ventilation (VE) against VCO_2_. Panel 5 is plot of VCO_2_ and HR against VO_2_. Panel 6 is plot of ventilatory equivalent for VO_2_ (VE/VO_2_) and VCO_2_ (VE/VCO_2_) against time. Panel 7 is plot of tidal volume against minute ventilation, with resting maximum voluntary ventilation on the X-axis and inspiratory capacity and vital capacity, measured at rest, on the Y-axis. Panel 8 is plot of gas exchange ratio (RER) against time. Panel 9 is plot of end tidal pO_2_ (P_ET_O_2_), end tidal pCO_2_ (P_ET_CO_2_) and pulse oximeter arterial oxyhemoglobin saturation against time. The normal subject (figure 2a) is a 59 year old female with scleroderma. Peak VO_2_ and *AT* are normal (panels 3 and 5) There are no signs of impaired oxygen flow, or ventilation/perfusion mismatching during exercise. Peripheral oxyhemoglobin saturation does not decrease during exercise. There is adequate breathing reserve. The subject with suspected pulmonary vasculopathy (figure 2b) is a 37 year old female with scleroderma. Peak VO_2_ and *AT* are reduced (panel 3, panel 5). Ventilatory equivalents are elevated and decrease only slightly during exercise (panel 6). End-tidal pCO_2_ is low and decreases during exercise (panel 9), consistent with reduced gas exchange efficiency rather than voluntary hyperventilation (RER is normal, panel 8). The patient stopped exercise because of leg pain. Four arrows are placed on each of Figures 2a and 2b that correspond to the branch-points described in Figure 1, Arrow 1 points to the peak VO_2_ in panel 3 (branch-point 1). Arrow 2 points to the *AT* in panel 5 (branch-point 2). Arrow 3 points to the VE/VCO_2_ at the *AT* in panel 6 (branch-point 3). Arrow 4 points to the changing P_ET_CO_2_ from start of exercise to *AT* in panel 9.

Figure 2 shows how the 15 variables taken from the CPET 9-panel plots of two representative SSc patients were analyzed. Figure 2a shows an SSc patient with a normal CPET response; Figure 2b shows another SSc patient with PV. The 4 arrows in Figures 2a and 2b correspond to the 4 branch-point parameters shown in Figure 1. The legend for figure 2 provides further detail.

Table 2 shows the 6 minute walk distance, key pulmonary function measurements, the presence of restrictive lung disease, pulse oximetry, and seven CPET parameters by diagnostic categories. Six patients achieved their predicted peak VO_2_, and another six stopped exercise prematurely without evidence of cardiovascular or pulmonary limitation. All 12 had linear increases in HR vs VO_2_ relationship towards their predicted value, normal *AT*, O_2_-pulse, and VE/VCO_2_ @ *AT*, as exemplified in figure 2a. We classified all 12 as normal. The other 16 patients achieved a symptom-limited test below their predicted peak VO_2_, and also had additional abnormalities. Of these 16, 5 were classified as LVD and 11 were classified as PV. Two of the latter also had significant restrictive lung disease with reduced FVC, TLC and DL_CO_. No patients were limited by obstructive lung disease, all had an adequate breathing reserve at peak exercise. In the patients who underwent HRCT due to clinical suspicion of interstitial lung disease (ILD), presence of ILD was found among all groups, with a trend to higher occurrence in the PV group. However, this difference did not reach statistical significance (p = 0.07).

**Table 2:** Physiologic measurements related to resting lung function and gas exchange during exercise in 28 scleroderma patients.

		Normal exercise capacity (NL)(n = 12)	Left Ventricular Dysfunction(LVD)(n = 5)	Pulmonary vasculopathy(PV)(n = 11)	NL vs. LVD	p-valueNL vs. PV	LVD vs. PV	
**Aerobic** **capacity**	6-MWD (m)	444±78	394±66	351±76	0.22	0.01		0.31
	Peak V̇O_2_(% predicted)	73.5±13.1	46.9±5.8	48.8±12.0	<0.001	<0.001		0.76
	*AT*(% predicted)	102.0±17.8	66.0±11.5	71.5±19.4	<0.001	<0.001		0.58
**Cardiac** **Function**	Peak O_2_ pulse(% predicted)	87.1±13.1	65.5±6.5	72.6±17.6	0.009	0.03		0.37
	Δ V̇O_2_/ΔWR ((ml/min)/W)	9.1±0.9	7.2±0.9	6.5±2.0	0.001	<0.03		0.45
**Ventilatory inefficiency**	**V̇E/V̇CO_2_** ***AT*** *	**29.8**±**2.9**	**30.2**±**2.4**	**39.2**±**8.3**	**0.87**	**<0.001**		**0.002**
	**P_ET_CO_2_** ***AT*** * **(mmHg)**	**37.9**±**4.5**	**37.4**±**4.0** *	**31.0**±**2.5** *	**0.82**	**<0.001**		**0.004**
	**Difference P_ET_CO_2_** ***AT*** ** – P_ET_CO_2_Start (mmHg)**	**3.2**±**2.3**	**+3.9**±**2.0** *	**−1.3**±**2.6** *	**0.93**	**<0.001**		**0.002**
**Lung function/** **imaging**	FVC(% predicted)	94.9±13.8	92.1±23.1	75.7±18.5	0.75	0.01		0.08
	FEV_1_/FVC(% predicted)	95.5±6.6	91.0±8.6	95.4±8.2	n/s (p = 0.60)			
	DL_CO_(% predicted)	89.8±22.6	71.8±12.9	54.7±17.6	0.18	<0.001		0.05
	FVC/DL_CO_(no unit)	1.14±0.17	1.30±0.28	1.45±0.29	0.17	0.01		0.37
	Presence of ILD	3/12	2/5	8/11	n/s (p = 0.07)			
**Pulse oximetry**	Resting SpO_2_(%)	96.8±1.81	95.8(2.17	96.5(2.07	n/s (p = 0.82)			
	Nadir SpO2(%)	91.9(5.52	94.4(3.78	90.8(6.14	n/s (p = 0.48)			

* = p<0.05, left ventricular dysfunction group vs. pulmonary vasculopathy group.

None of these patients showed signs of PVOD.

### Exercise Capacity

The cause of exercise limitation discerned from all 28 interpretable cardiopulmonary exercise tests was determined using the algorithm shown in Figure 1. Its branch-points systematically examined each of the key parameters from the 9-panel plots (Figure 2). Figure 2 shows where the branch-point data were obtained in each patient's 9-panel plot. Exercise capacity was significantly reduced due to identifiable defects in 16 of the 28 patients. In these patients, peak VO_2_ and VO_2_ at *AT* were <75% of the absolute predicted value and/or oxygen pulse reached a plateau at a significantly reduced value above the *AT* (Figure 2b, panel 2).

Several measurements in Table 2 are of special interest. FVC values were mildly reduced, 6MWD was moderately reduced, and DL_CO_ values were markedly reduced from normal in the PV group (p<0.001). However, reductions were qualitatively similar in the two cardiovascular disorders, the difference did not reach statistical significance. The FVC/DLCO ratio showed the same results: Only the normal and the PV group showed a difference which reached statistical significance (p = 0.01). The difference between the normal and the LVD group, as well as the difference between the PV and the LVD group were not statistically significant (p = 0.17 and p = 0.37, respectively).

Peak VO_2_, *AT*, peak O_2_ pulse and Δ VO_2_/ΔWR were all reduced in patients with PV and LVD, but the magnitudes and patterns of these reductions did not distinguish the two disorders. As single parameters, only PETCO2@AT (p = 0.004), VE/VCO2@AT (p = 0.002) and the changes in P_ET_CO_2_ from early exercise to the *AT* (p = 0.002) distinguished LVD from PV.

**Figure 3:**
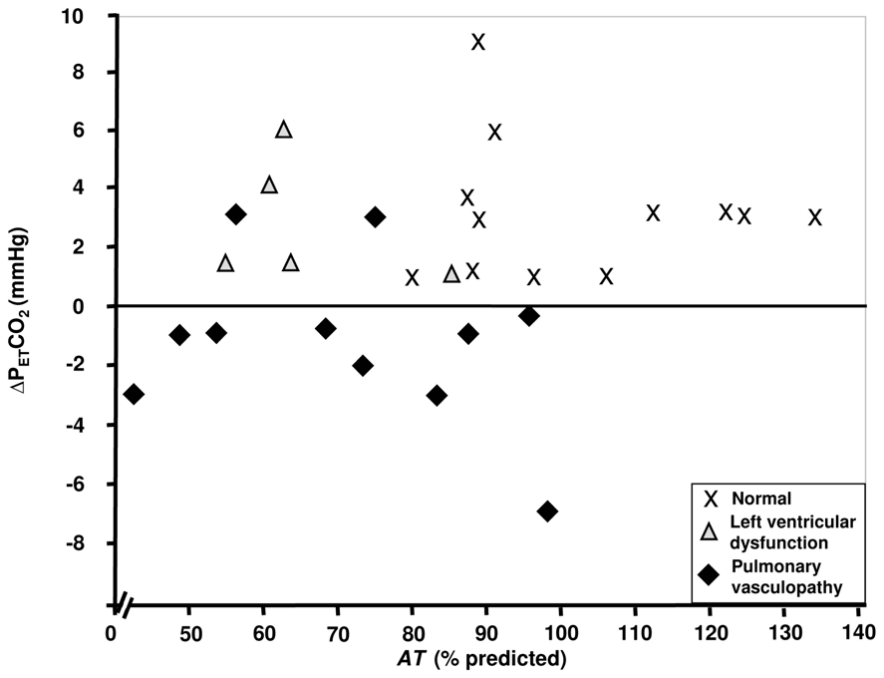
Difference between P_ET_CO_2_ at *AT* and P_ET_CO_2_ at start of exercise, plotted against *AT*, percent predicted, for SSc patients with normal exercise tolerance, left ventricular dysfunction, and pulmonary vasculopathy.

The directional change in P_ET_CO_2_ at the start of exercise to the *AT* (ΔP_ET_CO_2_) tends to be negative (decreases to the *AT*), as has been previously shown in patients with idiopathic PAH [17], [18]. In contrast, P_ET_CO_2_ increases from the start of exercise to the *AT* in the normal subjects and the patients with LVD (Fig. 3). There was no significant difference between the normal (3.2±2.3 mm Hg) and LVD (3.9±2.0) groups (p = 0.93) in the P_ET_CO_2_ change. However, the PV group (−1.3±2.6) differed significantly from both (p<0.001 and p = 0.002, respectively).

**Figure 4:**
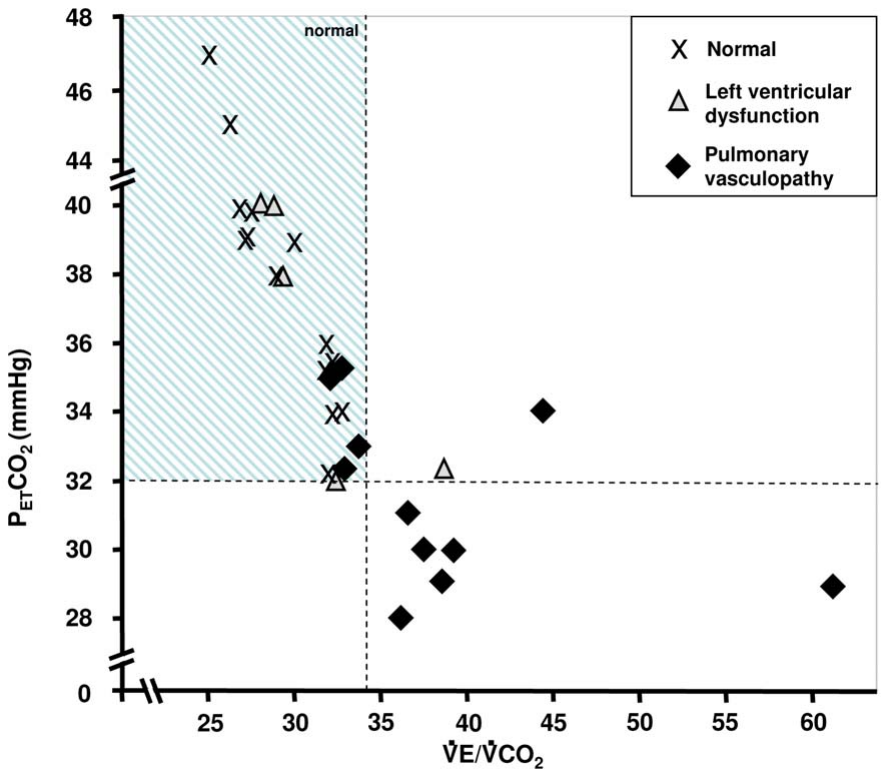
P_ET_CO_2_ as a function of VE/VCO_2_ at the anaerobic threshold in 28 SSc patients.

Figure 4 shows the relationships of P_ET_CO_2_ to VE/VCO_2_ at the *AT* of all patients. Although there is some overlap, most patients with pulmonary vasculopathy had a lower P_ET_CO_2_ and higher VE/VCO_2_ than the normal and LVD groups.

**Figure 5:**
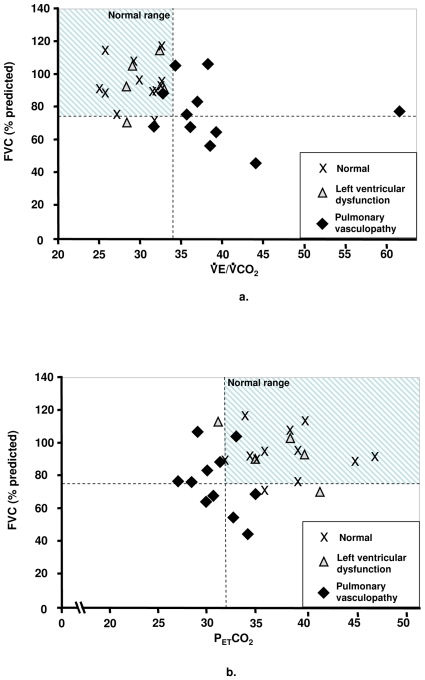
FVC as a function of P_ET_CO_2_at *AT *(Fig. 5a) and P_ET_CO_2_ at the *AT*(Fig. 5b) in 28 SSc patients with normal exercise tolerance, left ventricular dysfunction, and pulmonary vasculopathy.

The FEV_1_/FVC ratio was normal in all subjects. However, on average, our patients with PV tended to have lower FVC than the normal and LVD groups, (Table 2). To distinguish those patients with PV and restriction from those without or less restriction, we plotted the FVC against VE/VCO_2_ and P_ET_CO_2_ at *AT* (Figures 5a and 5b). Approximately half of the patients with ventilation-perfusion mismatch (high VE/VCO_2_ and low P_ET_CO_2_ at the *AT*) had significant reductions in FVC, while the others had PV with no or minimal restriction (normal FVC).

## Discussion

Previous studies have described lung gas exchange abnormalities at rest and during exercise in SSc patients [20]. However, this is the first study to show that abnormal gas exchange patterns during exercise, characteristic of PV, can be seen in patients with SSc without elevated pulmonary artery pressure on echocardiography or of having pulmonary vascular disease based on clinical suspicion. These abnormal CPET patterns may represent early PV, which with time, may lead to clinical and symptomatic pulmonary hypertension at rest.

Using CPET, we found evidence of possible PV in an SSc patient population, which was asymptomatic for the disease. A decreased peak VO_2_ along with a reduced *AT* has been the primary marker of reduced exercise capacity in patients with cardiovascular limitations to exercise [21]. However, patients with multi-organ diseases like scleroderma, are frequently exercise-limited with unclear cause. The 6MWD cannot be expected to define pathophysiology or differentiate causes of reduced exercise capacity. Therefore assessment of measures beyond 6MWD and peak VO_2_ measurements is needed to identify the specific pathophysiology underlying exercise intolerance.

In this study, we hypothesized that measures of ventilatory efficiency, specifically P_ET_CO_2_ and VE/VCO_2_ and their patterns of change during exercise, added to other gas exchange measures evaluating peak and sustainable cardiac output, or VO_2_, could be used to differentiate patterns which indicate possible PV from other causes of exercise gas exchange abnormalities. Elevated VE/VCO_2_ values at the *AT* or VCP are important non-invasive measurements of ventilation-perfusion mismatching due to loss of pulmonary vasculature, and can be identified without maximal exercise. The additional finding of a low PETCO2@AT was even more discriminatory when used to differentiate early LVD from early PV (Table 2).

Because of loss of perfusion to ventilated lung, there is less CO_2_ laden blood to release CO_2_ to the airspaces for a given ventilation in patients with suspected PV. Thus, to eliminate the metabolic CO_2_, ventilation must increase resulting in an elevated ratio of VE to VCO_2_. In left ventricular failure, it is also common for portions of the lung to be well-ventilated, but be poorly perfused. Thus, VE/VCO_2_ is commonly used as an index of the severity of LVD [22], [23]. Due to its pathogenesis, VE/VCO_2_ should invariably be increased in patients with PV [18], [24], [25]. Because of the loss of vascularity to lung acini, P_ET_CO_2_ is diluted in proportion to the fraction of underperfused acini. Thus, P_ET_CO_2_ is decreased as VE/VCO_2_ is increased, the degree depending on disease severity. In less severe stages of pulmonary vascular disease, small increases in VE/VCO_2_ are accompanied by large decreases in P_ET_CO_2_ [18] (Figure 4). Thus, a reduced P_ET_CO_2_ at the *AT* or VCP is a valuable marker of blood vessel loss, and may be sensitive in detecting early pulmonary vascular disease.

It has also been shown, in the transition from the start of exercise to the *AT*, that P_ET_CO_2_ tends to decrease in PAH, whereas the P_ET_CO_2_ tends to increase in LVD [17]. This observation appears to occur in SSc patients with suspected PV as well.

Figure 5 relates the degree of lung restriction (reduced FVC) to the elevation of VE/VCO_2_ (Fig. 5a) and dilution of PCO_2_ (Fig. 5b) at the *AT* or VCP. All three groups had reductions in FVC, but it is mainly the PV group that had the abnormally high VE/VCO_2_ and low P_ET_CO_2_ values. This might become therapeutically relevant in patients with SSc and borderline pulmonary hypertension. Presumably, the best candidates for specific therapy would be those patients with the highest VE/VCO_2_ and lowest P_ET_CO_2_ values and least lung restriction. However, the validation of this hypothesis is subject to further studies.

### Study limitations

Our diagnostic algorithm categorizing exercise pathophysiology, based on patterns of exercise gas exchange, was designed to identify scleroderma patients with characteristic patterns of PV and normal pulmonary artery pressure on echocardiography. We did not perform right-heart catheterization in our patients, as the study aim was to detect patterns of early PV in patients who might not have yet progressed to clinical resting pulmonary hypertension, so that an elevated PAP during a resting right heart catheterization might not have been evident, given a normal systolic PAP on echocardiography. Only long-term longitudinal evaluation of these patients will enable us to discern the rates of progression of these abnormalities, and may provide insight into the natural course of PV in patients with SSc.

True dead space/tidal volume ratio can only be calculated using arterial blood gas measurements. We did not do arterial blood sampling during exercise in order to avoid discomfort, and the potential for sudden peripheral vasospasm in SSc patients. However, increased VE/VCO_2_ beyond that found in normal subjects [26], and simultaneously decreased P_ET_CO_2_ at the *AT*, as well as specific changes in the patterns of these two variables as work rate is increased, strongly suggest that dead space ventilation is increased.

Although more patients with systemic sclerosis suffer from the limited type than from the diffuse type, the distribution between diffuse and limited SSc may have been shifted towards patients with the limited form of the disease in our cohort, as only a few patients were found to have the diffuse form. Thus, our findings might be influenced by an overrepresentation of patients with the limited form of SSc.

The differential diagnosis of pulmonary veno-occlusive disease (PVOD) in SSc patients, an important clinical question, is challenging. We could not definitely exclude PVOD in our subjects, as this would require histological confirmation. However, this procedure is not recommended as it carries a significant risk [27]. HRCT, which was performed in all patients with suspicion of ILD (20 out of 30 patients) did not show any findings consistent with PVOD such as enlarged mediastinal lymph nodes, alveolar hemorrhage, or septal lines in any of the patients. In the remaining 10 patients, HRCT was not indicated as clinical status, PFT, chest x-ray and (except for one asymptomatic patient) VE/VCO_2_ were normal, and hence the probability of PVOD is considered very low. Furthermore, in these patients the nadir SpO_2_ during exercise were significantly higher than the values found in PVOD patients reported by Montani et al [28].

We conclude that routine CPET may be a sensitive method to detect developing exercise intolerance and provide additional information on the mechanism of exercise limitation in SSc. More detailed analysis of the specific pathophysiological mechanism underlying the developing exercise intolerance, such as PV and LVD, might clarify the treatment direction and therefore might help in preventing progression. However, there are no data to prove this, and further investigations are warranted.
